# Cognitive Tunneling in Obstetrics and Gynecology: A Critical Review of Implications for Clinical Practice and Mitigation Strategies

**DOI:** 10.3390/diseases14090315

**Published:** 2026-08-28

**Authors:** Dan Boitor, Mihai Surcel, Cristina Ioana Rotar, Gheorghe Cruciat, Georgiana Nemeti, Andreea Florian, Daniel Muresan, Mihaela Oancea

**Affiliations:** 1 Department of Obstetrics and Gynecology, “Iuliu Hatieganu” University of Medicine and Pharmacy, 400012 Cluj-Napoca, Romania; dan.boitor@umfcluj.ro (D.B.); cristina.rotar@umfcluj.ro (C.I.R.); gheorghe.cruciat@umfcluj.ro (G.C.); georgiana.nemeti@umfcluj.ro (G.N.); andreea.florian@umfcluj.ro (A.F.); daniel.muresan@umfcluj.ro (D.M.); mihaela.oancea@umfcluj.ro (M.O.); 2 Department of Obstetrics and Gynecology, Emergency County Hospital Cluj-Napoca, 400006 Cluj-Napoca, Romania

**Keywords:** cognitive tunnelling, tunnel vision, attention tunneling, obstetrics and gynecology, mitigation strategies

## Abstract

Cognitive tunnelling poses significant risks in OB/GYN, where rapid clinical deterioration and time-pressured decision-making are common. This critical review synthesized evidence from a comprehensive literature search across multiple databases, yielding 414 unique papers examining cognitive tunnelling mechanisms, patient safety consequences, and mitigation strategies in obstetric and gynecological contexts. Medicolegal analysis reveals that cognitive tunnelling contributes to malpractice exposure and that adoption of structured safety programs addresses root causes and strengthens the clinical record for forensic review. Mitigation strategies were critically evaluated: metacognition and individual cognitive forcing strategies such as DECLARE (Differential, Examine, Consider, List, Assess, Review, Evaluate) and Pivot-and-Cluster show theoretical promise but depend on intact executive capacity that is depleted under the very conditions that precipitate tunnelling; debriefing demonstrates consistent improvements in team situational awareness and latent threat identification but requires organizational just culture and psychological safety; and hierarchical closed-loop communication systems provide the strongest evidence of reducing preventable adverse events by distributing attentional load across teams, though authority gradients risk suppressing junior-staff dissent. Despite growing recognition, significant knowledge gaps remain regarding measurement methodologies, obstetric-specific research, intervention effectiveness, and the integration of emerging artificial intelligence technologies. Addressing cognitive tunneling through quality studies with patient-centered outcomes and validated measures is essential for reducing medical errors and enhancing patient safety in women’s healthcare.

## 1. Introduction

Cognitive tunneling (CT), also referred to as tunnel vision or attention tunneling, constitutes a fundamental limitation of human information processing with substantial implications for medical practice [1]. The obstetrics and gynecology (OB/GYN) environment precipitates this specific failure mode. A specialty-specific risk profile with medicolegal consequences occurs, which cannot be adequately addressed by generic cognitive bias frameworks designed for slower-paced diagnostic reasoning [1,2]. Courts and expert witnesses routinely conflate CT with negligence precisely because no authoritative, focused evidentiary synthesis exists to distinguish the two, a gap that only a dedicated review can close [3,4]. Taken together, the unparalleled precipitating conditions of the OB/GYN setting, the disproportionate and irreversible harm to two simultaneous patients, the inadequacy of existing generic countermeasures, and the urgent medicolegal need for an operationalized evidentiary framework collectively constitute a compelling reason for treating CT in OB/GYN as a first-class subject of dedicated scholarly review.

Recent analyses of obstetric morbidity and mortality have identified cognitive bias, including CT, as a notable contributor to preventable harm [2,5,6]. Recognition of the impact of CT is therefore a prerequisite for designing mitigation strategies and delivering safer, more effective care to women and newborns.

This review synthesizes the current evidence on CT in OB/GYN and proposes mitigation strategies that reduce diagnostic error and strengthen patient safety. It further addresses medicolegal implications, current research, future directions, and the limitations and challenges that characterize the field.

## 2. Methods

This review employed a critical synthesis approach to examine CT in OB/GYN. The review was conducted between January 2026 and June 2026. Google Scholar, PubMed, and Zotero (Digital Scholar, Falls Church, VA, USA) were systematically searched. The search terms were: (“cognitive tunnelling” OR “cognitive tunnel” OR “tunnel vision” OR “attention tunnelling” OR “attentional narrowing” OR “cognitive bias” OR “diagnostic error”) AND (“obstetrics” OR “gynecology” OR “gynecology” OR “maternal-fetal medicine”). Inclusion criteria were studies regarding healthcare professionals working in OB/GYN or maternal–fetal medicine settings with mitigation strategies clearly specified. Studies were excluded if they met any of the following criteria: research conducted exclusively in non-healthcare contexts (aviation; military context); the non-peer-reviewed grey literature without methodological rigor; studies that mentioned CT only tangentially without substantial analysis or data. Given the search strategy and the inclusion of diverse study designs, strict adherence to PRISMA was not feasible. Despite this limitation, the methodology employed was rigorous, transparent, and appropriate for the breadth and complexity of the review question.

## 3. Results

The search yielded 414 unique papers after deduplication. Emphasis was placed on publications from 2000 to 2026, reflecting the modern era of patient safety science and dual-process theory applications in medicine. After a comprehensive analysis, 82 references were included in the review.

## 4. Discussion

### 4.1. Cognitive Architecture and Dual-Process Theory

**Figure 1:**
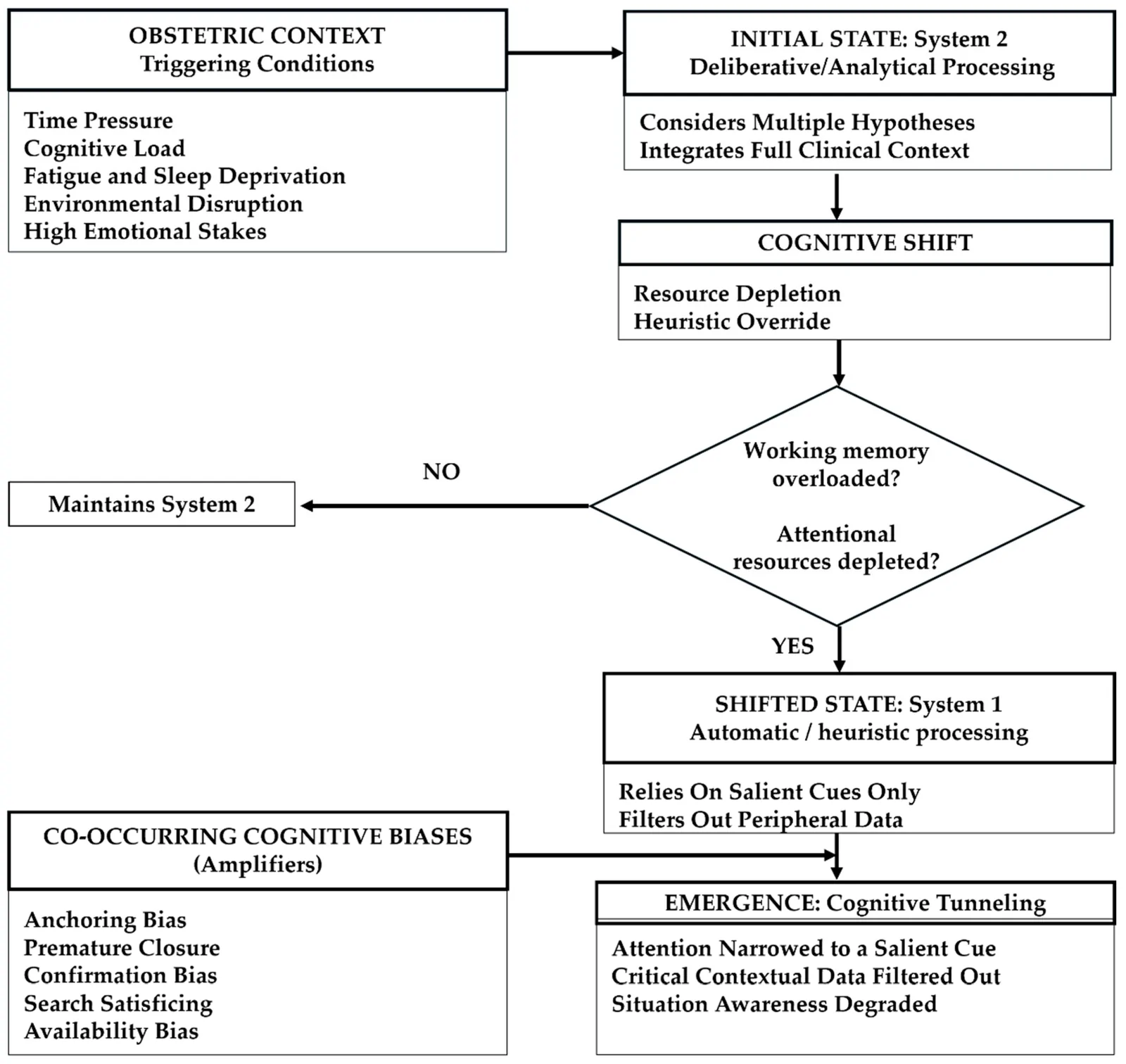
Flowchart of psychological mechanisms that lead to a cognitive shift and the emergence of cognitive tunneling, including examples from the obstetric setting.

The mechanism of CT involves allocation of limited attentional resources to a salient feature or task while filtering out potentially relevant peripheral information [7]. CT emerges from the architecture of human cognition, particularly the interaction between fast, reflexive processing (System 1) and slower, deliberative reasoning (System 2) described in dual-process theory [8,9,10]. Under high cognitive load, time pressure, and stress, clinicians shift toward System 1 processing, which, although efficient, is more susceptible to bias and selective attention [1]. This shift occurs through a discreet, stress-mediated suppression of prefrontal executive control that forces a wholesale transition from System 2 to System 1 processing (Figure 1). By this mechanism, the CT impairs not merely the weighting of available information but the very registration of it—a qualitative distinction that broad umbrella reviews systematically fail to capture with the granularity required for targeted countermeasure development [11,12].

Cognitive tunneling frequently co-occurs with other cognitive biases that compound the risk of diagnostic error [6,13,14,15,16,17,18,19,20], called amplifiers (Figure 1). These heuristics are particularly problematic in obstetrics, where multiple pathophysiologic processes may evolve simultaneously.

The HALT construct—an acronym denoting the physiological and affective states of Hunger, Anger (or Anxiety), Lateness (i.e., running behind, with associated stress), and Tiredness—represents a well-recognized framework in patient safety science for characterizing the internal conditions under which executive cognitive function is most severely compromised.

The theoretical basis for inclusion is compelling: each component of the HALT state directly depletes the prefrontal executive resources that underpin System 2 deliberative reasoning. Hunger and fatigue reduce sustained attentional capacity and increase reliance on heuristic, automatic processing. Anger and anxiety heighten amygdala-mediated threat responses that further suppress prefrontal regulatory control. The stress of running late in a complex clinical environment generates precisely the time-pressure conditions shown by Kahneman [11] and Croskerry [21] to accelerate the shift from reflective to reflexive cognition. When a clinician presents to an obstetric emergency already in a HALT state, the attentional threshold at which CT is triggered is substantially lowered, meaning that even moderately high-acuity scenarios—which a rested, normo-glycemic, emotionally regulated clinician might navigate analytically—become sufficient to induce pathological CT. This interaction is not additive but synergistic: HALT states and time pressure compound one another, each amplifying the cognitive cost of the other.

The additional dimension introduced by dynamic pathology is equally important and deserves separate treatment. Unlike static diagnostic problems, obstetric emergencies such as evolving placental abruption, ascending hemorrhagic shock, escalating severe pre-eclampsia, or the rapid sequence of complications that can follow shoulder dystocia present a continuously shifting clinical picture that demands iterative reassessment. A clinician in a HALT state, already tunnelling on the initial presenting feature, is particularly ill-equipped to track this dynamic evolution. The attentional resources required to update a mental model in real time are precisely those depleted by HALT, meaning that the clinician’s internal representation of the patient’s condition progressively diverges from clinical reality as the pathology evolves. This mechanism—tunnelling on an outdated, static representation while the patient deteriorates along a different trajectory—represents one of the most dangerous manifestations of the phenomenon this article addresses.

### 4.2. Implications of Cognitive Tunneling in Practice (Figure 2)

Cognitive tunneling is a major contributor to diagnostic errors in OB/GYN practice [22,23]. In obstetrics, tunneling-related errors may manifest as delayed recognition of fetal compromise during labor, missed maternal complications during delivery, or failure to identify evolving emergencies such as uterine rupture or amniotic fluid embolism [24]. Gynecologic practice shows distinct error patterns, including missed concurrent pathology during surgical procedures, delayed recognition of malignancy during routine procedures, and failure to identify complications during minimally invasive surgery [25].

**Figure 2:**
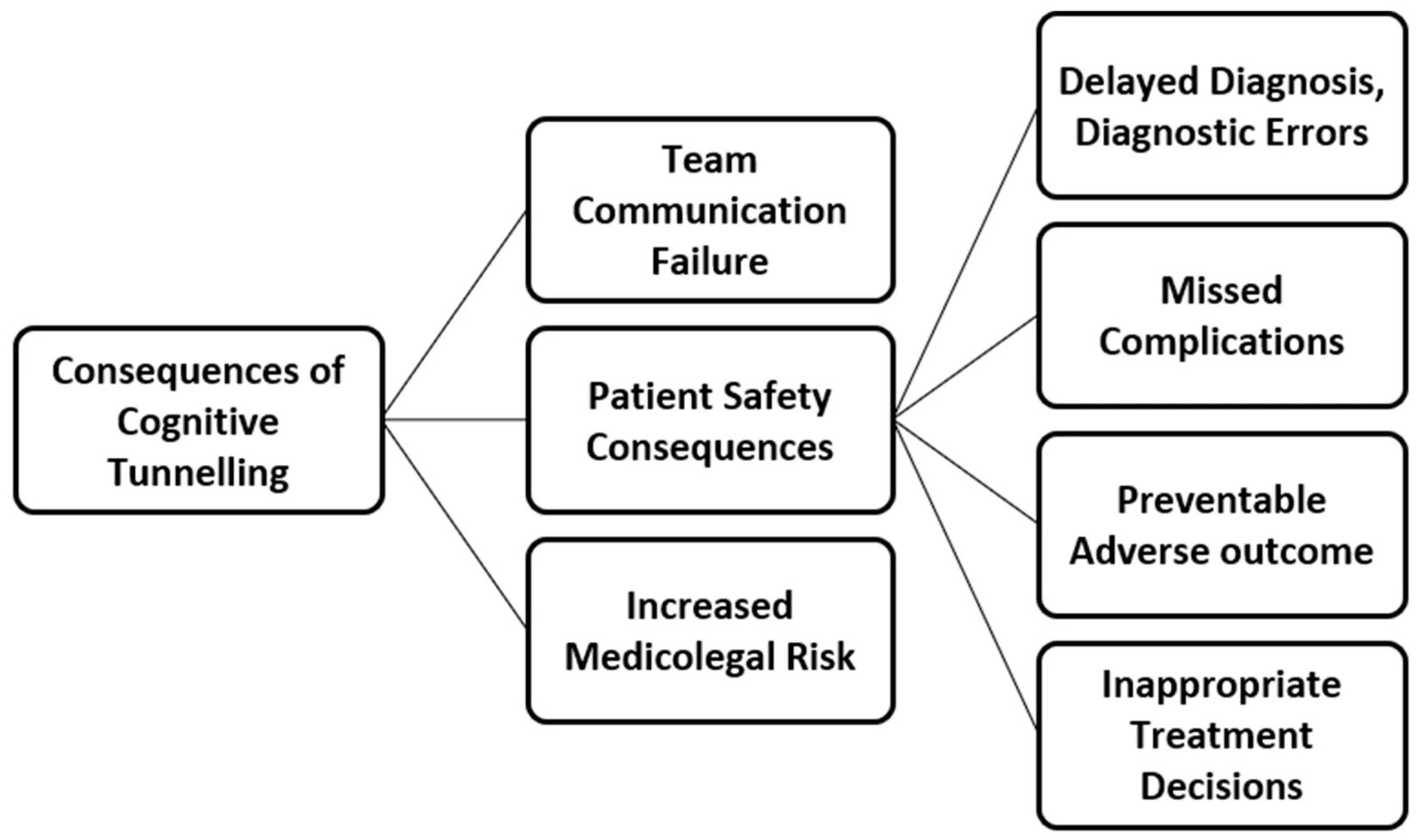
Implications of cognitive tunneling in practice.

Cognitive tunneling affects not only individual decision-making but also team functioning and communication in OB/GYN settings [26]. When team leaders experience CT, they may underutilize the knowledge and observations of their team members. Junior team members may hesitate to voice observations or concerns that contradict the apparent focus of senior clinicians, even when such input could prevent errors. Communication failures linked to CT also occur during handoffs and transitions of care, when important contextual information fails to transfer because of a narrow focus on specific aspects of patient status [27]. This is particularly consequential in obstetric settings, where care frequently transitions between providers and teams during labor and delivery.

Cognitive tunneling and related diagnostic errors carry significant medicolegal implications in OB/GYN practice [28,29], occupying a complex position at the intersection of human cognitive limitation and professional accountability. Malpractice claims in these specialties frequently involve allegations of failure to recognize or respond appropriately to clinical signs and symptoms, many of which may be attributable to cognitive bias, including CT.

In expert review and litigation contexts, the HALT state of the clinician at the time of the adverse event is increasingly recognized as relevant contextual evidence, though its legal weight requires careful delineation. Expert witnesses and courts do not reduce the objective standard of care on the basis of a clinician’s fatigue or hunger—the duty owed to the patient remains constant irrespective of the clinician’s internal state. However, establishing that a clinician was operating under HALT conditions at the time of the alleged error performs two distinct medicolegal functions. First, it provides a mechanistic account of how a competent clinician could have made the error in question—replacing the inference of negligence or incompetence with a scientifically grounded explanation of cognitive failure under physiological stress. This may be material to the assessment of culpability and to the distinction between system failure and individual deviation from standard of care. Second, and of greater systemic importance, the recurrent demonstration of HALT-state vulnerability in adverse event analyses provides the evidentiary basis for institutional and organizational liability. If a healthcare institution’s rota design, rest-break policies, staffing levels, or escalation protocols routinely place clinicians in HALT states during high-acuity clinical periods, the institution may bear a share of medicolegal responsibility for the resulting adverse outcomes that extends beyond the individual clinician. This reframing—from individual cognitive failure to system-generated vulnerability—is consonant with the high-reliability organization literature and with the trajectory of UK maternity inquiries, including the Ockenden Report [30] and the East Kent Review [31], both of which identified workforce and cultural factors, not merely individual clinical errors, as root causes of preventable harm.

Expert testimony in malpractice litigation increasingly recognizes the role of cognitive factors in medical error. Understanding these biases is relevant both for defense and for the ongoing education of medical professionals [32]. Courts and expert witnesses evaluate CT within rigorous frameworks that distinguish between understandable human limitations and deviations from accepted professional standards. The spectrum from excusable cognitive error to negligence is determined by whether the clinician maintained appropriate situational awareness within human cognitive limits, followed established protocols, sought consultation when appropriate, and documented clinical reasoning. Cognitive tunneling crosses into negligence when it leads to failures that a reasonably competent practitioner would have avoided—particularly failures to recognize high-risk features, failures to perform indicated confirmatory assessments, failures to escalate care when initial management is ineffective, and failures to follow established monitoring protocols.

Documentation and objective evidence play decisive roles in medicolegal outcomes, because retrospective review may expose patterns of attentional narrowing that contributed to adverse outcomes [33,34]. Contemporaneous records, electronic fetal monitoring (EFM), cardiotocography (CTG) traces, and multidisciplinary expert review form the evidentiary foundation for assessing whether cognitive tunneling contributed to a breach of care and whether that breach caused harm. However, the medicolegal reliance on CTG interpretation has been increasingly criticized, with current frameworks emphasizing that CTG analysis alone is insufficient to establish causation and that comprehensive, multidisciplinary expert review integrating clinical, biochemical, and neuroimaging evidence is essential.

Hindsight bias represents a pervasive threat to fair medicolegal analysis, with the post hoc ergo propter hoc fallacy leading to unjust attributions of causation to cognitive tunneling without adequate consideration of alternative etiologies. Good expert practice requires explicit acknowledgment of hindsight bias, distinction between what was knowable at the time and what is known in retrospect, and reliance on objective evidence rather than speculative reasoning [35].

The application of just culture principles in obstetric malpractice litigation remains limited, with an evidence gap regarding how formal just culture policies alter legal liability determinations [36]. The tension between individual accountability (required by the legal system) and system accountability (emphasized by patient safety frameworks) remains unresolved, creating challenges for implementing transparent error analysis and organizational learning while protecting clinicians from unfair liability [37].

Ultimately, addressing CT in OB/GYN practice requires balancing the recognition that cognitive biases are inherent human limitations with the professional responsibility to maintain standards of care that protect patients from preventable harm. This balance is best achieved through medicolegal frameworks that fairly distinguish between excusable human limitations and true negligence while promoting transparency, learning, and continuous improvement in patient safety.

### 4.3. Mitigation Strategies and Interventions (Table 1)

The main mitigation strategies and interventions proposed to address cognitive tunneling are summarized in Table 1. These interventions operate at multiple levels and are intended to facilitate the recognition of discordant or evolving clinical information, and reduce inappropriate persistence with an initial diagnostic hypothesis. Importantly, however, the evidence supporting these approaches is heterogeneous and, in several domains, remains limited, with relatively few studies specifically examining cognitive tunneling in obstetrics and gynecology. Accordingly, the interventions presented in Table 1 should be regarded primarily as potentially useful mitigation strategies rather than uniformly evidence-based solutions. Their effectiveness is likely to depend on the clinical context, the characteristics of the decision-making environment, and the extent to which interventions are integrated into broader systems for patient safety, teamwork, and diagnostic error reduction.

**Table 1:** Synthesis of Cognitive Tunneling Mitigation Strategies Across Three Intervention Levels.

Level	Mitigation Strategies
Individual	Metacognitive Training Programs Cognitive Forcing Strategies Simulation-Based Education Reflective Practice and Cognitive Autopsy Mindfulness and Attention Training
Team-Based	Structured Communication Protocols Cross-Checking and Verification Procedures Role-Defined Cognitive Aids Psychological Safety and Speaking-Up Culture Multidisciplinary Team Rounds and Case Review Structured Debriefing After Critical Events
Systemic/ Technological	Human Factor Redesign of Data Displays Contextualized Clinical Decision Support Systems Intelligent Alerting Systems with Context-Aware Design Workload Management and Staffing Optimization Standardized Protocols and Clinical Pathways Organizational Culture of Safety and Error Disclosure Cognitive Bias Assessment and Monitoring Tools Artificial Intelligence and Machine Learning Integration

#### 4.3.1. Educational Approaches

Education about cognitive biases, with raising awareness about CT, constitutes a fundamental component of mitigation in OB/GYN practice [38]. Continuing education programs incorporating cognitive bias training have shown promise for improving diagnostic accuracy and reducing errors [39,40,41]. These programs typically combine didactic instruction with interactive exercises and case discussions that help clinicians internalize bias recognition and mitigation.

Simulation-based education offers a particularly effective means of demonstrating and addressing CT in a controlled setting [42]. High-fidelity simulations can reproduce the conditions that promote CT while permitting structured debriefing and reflection on cognitive processes. This strategy has been shown to improve recognition of cognitive biases and to reduce their impact on clinical decision-making [39].

#### 4.3.2. Systematic and Procedural Interventions

Systematic approaches to reducing CT include structured decision-making protocols and checklists that compel consideration of multiple factors [39,43]. Cognitive forcing strategies are structured interventions designed to interrupt System 1 and to engage System 2. The DECLARE mnemonic (Differential, Examine, Consider, List, Assess, Review, Evaluate) provides a checklist that guides clinicians through systematic consideration of alternative diagnoses, critical appraisal of supporting and contradicting evidence, and explicit evaluation of cognitive biases that may be shaping their reasoning [44,45]. Each step enforces a deliberate pause, requiring documentation of differential diagnoses, reassessment of initial assumptions, and consideration of the question “What else could this be?”, thereby counteracting premature closure and anchoring bias [44,46]. The Pivot-and-Cluster strategy employs a two-phase approach: the pivot involves deliberately stepping back from an initial diagnostic impression to consider alternative hypotheses, while clustering organizes clinical information into meaningful patterns that may reveal overlooked relationships or contradictory evidence [47,48]. Both strategies share a common mechanism: introduction of structured decision points that disrupt CT and create opportunities for error detection and correction before clinical decisions are finalized.

Team-based interventions, including structured communication protocols and cross-checking procedures, can help identify and correct individual tunneling [49]. Standardized communication tools such as SBAR (Situation, Background, Assessment, Recommendation) ensure that relevant contextual information is shared and considered [50]. SBAR directly counteracts the communication fragmentation that CT produces, particularly at the critical juncture of clinical handover. A tunneled clinician transferring care is liable to communicate only the focal concern driving their narrowed attention, omitting contextual information that a receiving clinician would need to form an accurate situational assessment. SBAR’s mandatory structure compels the communicating clinician to verbalize background and contextual data even when their own attention has narrowed, thereby preserving informational completeness independently of the sender’s cognitive state.

The theoretical contribution of Modified Early Obstetric Warning Score (MEOWS) and Modified Early Warning Score (MEWS) to CT mitigation operates through the mechanism of cognitive offloading: by externalizing the continuous vital-sign monitoring function into a standardized, threshold-triggered scoring system, these tools relieve the clinician of the attentional burden of tracking multiple physiological parameters simultaneously, thereby reducing the cognitive load conditions that precipitate CT in the first instance [51]. Critically, the objective alert generated when a MEOWS or MEWS threshold is breached functions as a mandatory pattern-interrupt—an external cue that forces a shift from System 1 automatic processing back to System 2 deliberative appraisal, precisely at the moment when CT would otherwise cause deterioration to go unrecognized. The UK Confidential Enquiry into Maternal Deaths and Morbidity (MBRRACE-UK) has consistently identified failure to recognize and act on signs of deterioration as a recurrent contributory factor in preventable maternal deaths, a pattern directly consistent with CT; the adoption of MEOWS within UK’s National Health System (NHS) maternity units represents a systemic response to exactly this failure mode [52].

Timeout procedures before critical decisions or interventions create opportunities to step back from immediate focus and consider broader clinical context [53,54]. These brief pauses allow for reflection and for input from team members that might otherwise be overlooked owing to tunneling.

#### 4.3.3. Technological Solutions

Technology-based interventions offer promising approaches to mitigating CT through improved information presentation and decision support [55]. Monitoring and display systems that integrate multiple data streams and present contextual information can reduce tunneling by making relevant information more salient and accessible [56]. Clinical decision support systems that issue alerts or reminders about alternative diagnoses and risk factors can counteract tunneling by forcing consideration of factors that might otherwise be overlooked [57]. RAG-anchored guidelines employ a Red–Amber–Green color-coded threshold system to stratify clinical risk and embed escalation triggers directly into bedside documentation and institutional protocols. Their principal benefit lies in the standardization of deterioration recognition: by anchoring clinical action to objective, pre-specified physiological thresholds, RAG-based tools reduce the inter-clinician variability that inevitably arises when threshold judgements are left to individual clinical discretion, particularly under conditions of fatigue, high workload, or competing demands. In the obstetric context, RAG thresholds embedded within MEOWS have demonstrated value in prompting earlier escalation to senior review, and their relationship to CT mitigation is theoretically coherent. A color-triggered alert functions as an externally generated pattern-interrupt that forces attentional reorientation independent of the clinician’s own vigilance. Nevertheless, RAG tools carry limitations that should be addresses explicitly. The most significant of these is the risk that the color category itself becomes the focus of clinical attention rather than a prompt for comprehensive clinical reassessment. A patient scoring amber may receive a threshold-driven response rather than genuine diagnostic reappraisal, and green or amber categorization can generate false reassurance in presentations where deterioration is subtle, atypical, or progressing rapidly between scoring intervals. Furthermore, RAG thresholds have been validated predominantly on specific demographic and clinical populations, raising legitimate concerns about differential performance in high-body mass index patients, those with pre-existing cardiovascular or respiratory conditions, and women from non-white ethnic groups.

Black-box scores present a distinct and more technically complex set of considerations. These are AI-generated algorithmic risk scores whose predictive power derives from large-scale pattern recognition across multiple data streams but whose internal computational logic is not accessible to or interpretable by the clinician receiving the output. Their clinical benefit in the obstetric setting is potentially considerable: black-box hemorrhage prediction tools, sepsis recognition scores, and deterioration indices have demonstrated promising sensitivity in early prospective studies. When functioning accurately, they can generate a meaningful alert before a tunneled clinician’s narrowed attention has registered the threat from the full clinical picture. However, the limitations of black-box scores are sufficiently serious to warrant the sustained critical attention the reviewer requests. Most fundamentally, a clinician who cannot interrogate the reasoning behind a score cannot meaningfully evaluate it; thus, they cannot prompt the deliberative System 2 reassessment that genuine debiasing requires. The medicolegal implications of this dynamic remain unresolved: when a black-box score contributes causally to an adverse outcome, the distribution of liability between the clinician who followed the score, the institution that deployed it, and the developer whose training data may have been demographically unrepresentative or methodologically flawed is deeply contested and as yet inadequately addressed by existing regulatory frameworks.

A concern that applies with equal force to both tool types is that neither RAG-anchored guidelines nor black-box scores were designed to develop clinical reasoning capacity—they were designed to compensate for its known failure modes under stress. This compensatory function is genuinely valuable, but it is insufficient as a standalone safety strategy, because tools that systematically replace vigilance and independent reasoning risk eroding precisely the clinical skills that remain indispensable when those tools malfunction, produce false outputs, or are unavailable in resource-limited or emergency settings. We recommend that RAG-anchored tools be implemented alongside training explicitly designed to teach clinicians to treat color triggers as prompts for full clinical reassessment rather than as decision endpoints in themselves. On the other hand, black-box scores deployed in obstetric settings should be required to meet minimum explainability standards and undergo prospective validation in demographically representative cohorts.

Artificial intelligence (AI) and machine learning tools are increasingly being developed to support pattern recognition and diagnostic reasoning in ways that complement human cognition and may mitigate bias [58,59]. Such systems can potentially identify patterns or risk factors that human cognitive limitations would otherwise mask. Nevertheless, AI tools have significant drawbacks that must be addressed. The most clinically significant limitation of AI decision support is the phenomenon of automation bias—the tendency of human operators to over-rely on automated recommendations, accepting algorithmic outputs without adequate critical appraisal and discounting contradictory clinical evidence [60]. A clinician who tunnels on an AI recommendation may do so with an unwarranted sense of algorithmic authority, suppressing the very metacognitive doubt that independent clinical reasoning would have preserved. In obstetric settings, where AI tools are increasingly proposed for CTG interpretation, sepsis recognition, and hemorrhage prediction, the risk of automation bias warrants explicit acknowledgement and targeted mitigation—including training that emphasizes critical evaluation of AI outputs rather than passive acceptance.

Many contemporary AI systems employed in clinical decision support, particularly those based on deep learning architectures, generate recommendations without producing an interpretable account of the reasoning that produced them. This opacity creates a fundamental problem in the context of CT mitigation: a clinician cannot use an AI recommendation as a genuine cognitive forcing strategy if the recommendation is not accompanied by a transparent rationale that can be evaluated, accepted, or challenged. A black-box alert that a patient is at elevated risk of uterine rupture may prompt appropriate action; it cannot, however, support the kind of deliberative System 2 engagement that effective debiasing requires.

AI systems perform only as reliably as the datasets on which they were trained. In obstetrics, training datasets have historically over-represented certain demographic groups and clinical settings, raising legitimate concerns that AI tools validated in tertiary academic centers may perform less reliably in district general hospitals or that performance may vary across patient populations differing in ethnicity, body habitus, comorbidity profile, or gestational complexity. Where an AI tool performs less reliably for a specific subgroup, the clinician who defers to its output—on the mistaken assumption of universal validity—is exposed to a systematic, demographically structured error that the unaugmented clinical encounter might not have produced. This limitation has direct medicolegal implications: if an AI tool known to perform less reliably in certain populations is deployed without appropriate caveats, institutional liability may extend beyond the individual clinician to the organization that implemented the system.

The utility of any AI alert system as a cognitive interrupt depends on the clinician treating each alert as a meaningful signal warranting attentive response. Where AI systems generate high volumes of alerts with variable positive predictive values, clinicians rapidly develop the same habituation response observed in relation to conventional monitoring alarms, dismissing alerts reflexively rather than engaging with them analytically. This alert fatigue does not merely reduce the effectiveness of AI as a mitigation tool; it may actively consolidate tunnelling behavior by reinforcing the clinician’s disposition to filter out peripheral signals—precisely the disposition that CT exacerbates.

Professionally, the duty of care in obstetric practice is borne by the registered clinician, not by an algorithm; professional accountability cannot be delegated to a decision support system, and no AI recommendation, however well-validated, discharges the clinician’s independent duty to assess, reason, and act in the patient’s best interest. Regulatorily, the current frameworks collectively position AI as a tool that operates within, not in place of, clinical judgment. Cognitively, there is a credible risk that sustained over-reliance on AI decision support erodes the very clinical reasoning skills—pattern recognition, hypothesis generation, differential diagnosis construction—whose maintenance is essential for competent performance in the inevitable circumstances where AI support is unavailable, malfunctioning, or mistaken. The clinician who has habitually deferred to algorithmic guidance may, in those circumstances, be more vulnerable to CT, not less.

AI tools in obstetric settings should be designed, implemented, and evaluated explicitly as cognitive partners rather than as autonomous advisors whose recommendations are expected to be followed.

#### 4.3.4. Organizational and Cultural Interventions

A reduction in CT at the organizational level requires a culture in which team members at every level can voice concerns and contribute observations. Such a culture is supported by structured solicitation of input across the team and by the adoption of systematic, protocol-driven decision frameworks [61,62]. Leadership behavior is central to this process; targeted training that enables clinical leaders to recognize and mitigate their own cognitive biases reduces the conditions under which CT emerges.

Quality improvement initiatives designed to address cognitive contributors to medical error provide a systematic mechanism for organizations to confront CT and related biases [63,64]. Scheduled debriefing and structured reflection allow teams to identify episodes of CT and to develop preventive strategies [65]. Such programs typically incorporate cognitive analysis of adverse events and implementation of targeted countermeasures, and they may be embedded in routine practice or activated in response to defined triggers or outcomes.

Workload management and fatigue reduction address system-level contributors to CT [45]. Lowering cognitive load and improving working conditions create operational environments in which biased decisions and downstream errors are less probable [66].

#### 4.3.5. Critical Considerations upon Mitigation Strategies of CT in OB/GYN

Generic mitigation strategies are paradoxically ineffective against CT because tunnelling selectively impairs the executive and metacognitive capacities required to deploy those very tools. Despite their intuitive appeal and widespread advocacy, the evidence base for individual debiasing in OB/GYN is notably weak, characterized by small exploratory studies, simulation-based assessments with uncertain clinical transfer, and an absence of patient-outcome data. Metacognition and cognitive forcing strategies are grounded in dual-process theory but share a fundamental paradox: both require intact executive capacity that is depleted under the stress and time pressure that precipitate CT. Mitigation of CT necessitates a wholly different class of interventions, including structured re-anchoring protocols (DECLARE), Pivot-and-cluster strategies, and team-based cognitive forcing, whose evidence base is sufficiently mature to warrant standalone systematic synthesis [44,47].

The evidence base for team-based strategies is substantially stronger than that for individual interventions, with multiple studies demonstrating improvements in team performance. Debriefing demonstrates consistent improvements in team situational awareness and latent threat identification, but its effectiveness depends on organizational just culture and psychological safety. Hierarchical closed-loop systems provide the strongest evidence of reducing preventable adverse events by distributing attentional load across teams, though authority gradients risk suppressing junior-staff dissent. However, effects on patient outcomes remain inconsistent, and implementation barriers continue to limit real-world effectiveness.

Systemic and technological mitigation strategies aim to redesign the environment in which clinicians work, providing cognitive support through checklists, decision aids, clinical decision support systems, artificial intelligence-assisted monitoring, and organizational safety culture reforms. The evidence base ranges from moderate-quality simulation studies for cognitive aids to preliminary and largely simulation-based evidence for AI, with robust real-world outcome trials remaining scarce.

This critical discussion reveals a substantial gap between the theoretical appeal of cognitive bias-mitigation strategies and the evidence base supporting their effectiveness in OB/GYN. While multi-level frameworks are conceptually compelling, the current evidence is insufficient to guide confident implementation in many domains. No single strategy sufficiently mitigates cognitive tunnelling in OB/GYN. Randomized controlled trials with patient-centered outcomes and validated real-time measures of CT are urgently needed.

### 4.4. Current Research and Future Directions

Current research efforts focus on the development of reliable instruments for measuring cognitive bias in clinical environments [67]. Validated tools, including the Clinicians’ Awareness Towards Cognitive Errors (CATChES) questionnaire, permit assessment of both individual and organizational susceptibility to cognitive bias [18].

Simulation-based studies continue to characterize the prevalence and impact of CT in OB/GYN practice [68]. In parallel, real-world observational studies are increasingly deployed to determine how CT manifests in routine clinical care rather than in simulated conditions [69,70]. These observational investigations clarify the ecological validity of simulation findings and the effectiveness of interventions when applied in practice.

Evaluation of intervention effectiveness remains an active research domain, with studies assessing the impact of multiple strategies on the reduction in CT [70]. Implementation science methods are being applied to determine how bias-mitigation strategies can be deployed effectively in clinical settings [69,71]. Comparative effectiveness research has begun to differentiate among bias-mitigation approaches and to identify which strategies perform best in defined contexts [20]. This work is essential for the formulation of evidence-based recommendations for clinical practice.

The application of AI and machine learning to clinical decision-making remains under active investigation [72,73]. Current AI systems are oriented predominantly toward prediction, but systems specifically designed to counter CT are anticipated. Human–computer interaction research examines how clinical interfaces and displays can be configured to reduce CT while preserving usability and workflow efficiency [48,74]. Mobile and wearable platforms are being evaluated as delivery vehicles for bias-mitigation interventions and for real-time feedback on cognitive processes [75,76]. AI-based decision support may reduce some forms of cognitive bias, while it may also introduce automation bias (over-reliance on algorithmic recommendations), and new medicolegal challenges related to the responsibility for medical decisions.

Augmented reality systems may provide valuable intraoperative tools during OB/GYN surgery, including contextual safety reminders, visual complication alerts, anatomical guidance overlays, and workflow checkpoint prompts.

Real-time cognitive state monitoring through eye-tracking, physiological monitoring, voice analysis, and interaction analytics is intended to detect escalating cognitive load. Research progress in this domain is constrained by the requirement to achieve acceptable privacy safeguards and clinician trust [77].

An emerging concept is the cognitive digital twin of the clinician, a model intended to estimate workload, attention allocation, diagnostic confidence, and tunneling risk on an individual basis. Continuous tracking of a clinician’s cognitive state requires sensitive data acquisition and demands rigorous data governance and security frameworks [78].

### 4.5. Limitations and Challenges

Current research on CT in OB/GYN faces several limitations that constrain the strength of available evidence and resulting recommendations [79]. Many studies rely on simulation rather than real-world clinical environments, which raises concerns regarding the external validity of reported findings. Measurement of cognitive biases in clinical practice remains methodologically difficult, as direct observation of cognitive processes is rarely feasible and may itself perturb the process under study [80]. The multifactorial nature of medical error further complicates efforts to isolate the specific contribution of CT from other contributing determinants [81], challenging attempts to demonstrate the discrete effectiveness of targeted interventions.

Implementation of bias-mitigation strategies in clinical practice encounters numerous obstacles, including resistance to change and competing institutional priorities [71,82,83]. Clinicians may be skeptical of interventions perceived to question clinical judgment or to add procedural steps to already-congested workflows. The time and resource demands of comprehensive CT mitigation programs are substantial, creating barriers for healthcare organizations operating under constrained budgets [61,84].

Long-term follow-up studies are required to evaluate the durability of intervention effects. Sustaining behavioral change over time represents an additional challenge, as initial improvements frequently attenuate without ongoing reinforcement and structured support [61,85].

The implementation of CT mitigation strategies also raises ethical considerations regarding the balance between structured decision support and preservation of professional autonomy [71,83]. Interventions perceived as overly prescriptive, or as undermining individual clinical judgment, are likely to encounter resistance and to demonstrate reduced effectiveness in practice.

Privacy and confidentiality concerns arise particularly with technologies that monitor or analyze clinical decision-making processes [86,87]. These concerns must be addressed explicitly during the design and deployment of technological solutions.

Comprehensive evaluation of intervention effects is essential. The potential for unintended consequences must be carefully considered, as strategies designed to mitigate one form of bias may inadvertently introduce or amplify others [61,87,88].

### 4.6. Limitations of the Present Study

Several methodological limitations of the present review warrant explicit acknowledgement. As with all narrative syntheses, the selection and interpretation of evidence are susceptible to confirmation bias: the thematic framing adopted by the authors may have privileged studies congruent with the conceptual argument being developed, and the absence of a pre-registered protocol means that the search strategy and inclusion decisions were not fully insulated from post-hoc rationalization. Efforts were made to mitigate this risk through multi-database searching yielding 414 unique sources and independent verification of 20% of data extraction decisions; nevertheless, the subjectivity inherent in narrative synthesis cannot be entirely eliminated, and the findings should be interpreted accordingly.

No formal PRISMA-compliant screening flowchart was generated. Although PRISMA reporting standards were developed primarily for systematic and scoping reviews rather than critical reviews [89], the absence of a published flowchart documenting records identified, screened, assessed for eligibility, and included limits the transparency and reproducibility of the search process.

Most fundamentally, the field currently lacks validated, real-time instrumentation capable of objectively detecting CT as it occurs in live clinical settings. Existing measures rely predominantly on retrospective case analysis, simulation-based observation, eye-tracking in controlled environments, and post-hoc cognitive autopsy, none of which fully captures the phenomenology of tunnelling under genuine time pressure with real patient stakes. This instrumentation gap means that the causal claims advanced in this review—however well-supported by convergent indirect evidence—remain inferential rather than directly empirically verified and that effect-size estimates for mitigation strategies are largely derived from proxies such as team performance scores, diagnostic accuracy rates, and adverse event frequencies rather than from measures of tunnelling per se. Addressing this gap represents perhaps the most important methodological frontier for future research in this area.

## 5. Conclusions

Cognitive tunneling represents a significant threat to patient safety and quality of care in OB/GYN practice. Mitigation strategies address individual, team, system, and technological determinants in parallel.

Despite substantive progress in characterizing CT and developing mitigation strategies, important challenges persist. Research limitations and implementation barriers must be addressed concurrently to advance the field and improve patient outcomes.

As the field continues to evolve, sustained research and education will be required to address the complex challenges posed by CT and related cognitive biases. Future research should prioritize the development of validated methods for measuring CT in clinical practice and the evaluation of intervention effectiveness in real-world settings. Addressing CT through quality studies with patient-centered outcomes and validated measures is essential for reducing medical errors and enhancing patient safety in women’s healthcare.

## Data Availability

No new data were created or analyzed in this study. Data sharing is not applicable to this article.

## Abbreviations

The following abbreviations are used in this manuscript:

CT	Cognitive tunneling
HALT	Hunger, Anger (or Anxiety), Lateness, Tiredness
EFM	Electronic fetal monitoring
CTG	Cardiotocography
DECLARE	Differential, Examine, Consider, List, Assess, Review, Evaluate
SBAR	Situation, Background, Assessment, Recommendation
MEOWS	Modified Early Obstetric Warning Score
MEWS	Modified Early Warning Score
MBRRACE-UK	UK Confidential Enquiry into Maternal Deaths and Morbidity
NHS	National Health System
RAG	Red–Amber–Green
CATChES	Clinicians’ Awareness Towards Cognitive Errors
